# Live kidney donation: are concerns about long-term safety justified?—A methodological review

**DOI:** 10.1007/s10654-016-0168-0

**Published:** 2016-06-28

**Authors:** Shiromani Janki, Ewout W. Steyerberg, Albert Hofman, Jan N. M. IJzermans

**Affiliations:** 1 Department of Surgery, Division of HPB and Transplant Surgery, Erasmus MC, University Medical Center, Rotterdam, The Netherlands; 2 Department of Public Health, Erasmus MC, University Medical Center, Rotterdam, The Netherlands; 3 Department of Epidemiology, Erasmus MC, University Medical Center, Rotterdam, The Netherlands; 4 Department of Epidemiology, Harvard T.H. Chan School of Public Health, Boston, MA USA

**Keywords:** Live kidney donation, Methodology, Comparison studies, Follow-up, Outcomes, Safety

## Abstract

Live kidney donors are exhaustively screened pre-donation, creating a cohort inherently healthier at baseline than the general population. In recent years, three renowned research groups reported unfavourable outcomes for live kidney donors post-donation that contradicted their previous studies. Here, we compared the study design and analysis of the most recent and previous studies to determine whether the different outcomes were due to methodological design or reflect a real potential disadvantage for living kidney donors. All six studies on long-term risk after live kidney donation were thoroughly screened for the selection of study population, controls, data quality, and statistical analysis. Our detailed review of the methodology revealed key differences with respect to selection of donors and compared non-donors, data quality, follow-up duration, and statistical analysis. In all studies, the comparison group of non-donors was healthier than the donors due to more extensive exclusion criteria for non-donors. Five of the studies used both restriction and matching to address potential confounding. Different matching strategies and statistical analyses were used in the more recent studies compared to previous studies and follow-up was longer. Recently published papers still face bias. Strong points compared to initial analyses are the extended follow-up time, large sample sizes and better analysis, hence increasing the reliability to estimate potential risks for living kidney donors on the long-term. Future studies should focus on equal selection criteria for donors and non-donors, and in the analysis, follow-up duration, matched sets, and low absolute risks among donors should be accounted for when choosing the statistical technique.

## Introduction

Live donor kidney transplantation is the treatment of choice for patients with end-stage renal disease (ESRD). The benefits of this treatment include pre-emptive transplantation, superior organ quality, and increased graft survival [1] and have led to an increase in live kidney donations and consecutive transplants. Despite this increase, the growing demand for donor kidneys cannot be matched, which has led to an increase in the number of extended-criteria live donors with minor comorbidities, such as well-regulated hypertension or higher body mass index (BMI) [2]. As a result, more than 20,000 transplants from live kidney donors are performed annually worldwide, and this number has remained stable over the past decade [3, 4].

Live kidney donors are individuals who willingly undergo major surgery to improve the well-being of someone else. It is of the utmost importance to minimize risks, such as the intra-operative risk of bleeding [5, 6] and mortality [7], and maximize donor safety during and after donation as well as in the long-term. Live kidney donors are exhaustively screened by a multidisciplinary team of transplant professionals and anaesthesiologists prior to donation, resulting in a cohort that is inherently healthier at baseline than the general population. Therefore, selecting non-donors with baseline health similar to accepted donors is difficult and may affect estimates of any potential risks attributable to donation. In addition to the problem of adequate selection, an extended follow-up period for live kidney donors is important for revealing the risks of donation on their long-term health [8, 9].

Three renowned research groups recently uncovered unfavourable outcomes for live kidney donors following donation compared to non-donors, including an increased risk of cardiovascular and overall mortality [8], increased risk of ESRD [8, 9], and increased risk of gestational hypertension and preeclampsia [10]. The number of events and absolute risks are low. Previous publications from these research groups [Oslo University Hospital, Johns Hopkins Medical Institutions, and the Donor Nephrectomy Outcomes Research (DONOR) Network] did not demonstrate unfavourable outcomes detrimental to live kidney donors, as they reported a lower risk of long-term cardiovascular [11] and overall mortality [11–13] and lower risk of cardiovascular events [13]. This is remarkable because studies from the same research group largely included the same donor population. We compared the study design and analysis of the three most recent studies and the previous studies from the same research groups.

## Materials and methods

### Literature search

We searched for studies that reported negative outcomes following live kidney donation using MEDLINE, Embase, CENTRAL (the Cochrane Library 2013), OvidSP, and Google Scholar.

### Literature screening

We selected studies published in the last 5 years with an impact factor >15 or high citation rate >20. We found three studies by three different research groups [8–10]. Previously, studies from these research groups reported favourable outcomes following live kidney donation [11–13] in the same donor cohort. The discrepancies in outcomes of these studies have been highly debated within the transplant community.

### Outcome

In light of the impact of these studies on the transplant community, we compared the methodology used in the studies and the likely impact on outcomes. The six studies were thoroughly screened by two authors (SJ and JNMI) in regard to the selection of the study population, data quality, and statistical analysis.

## Results

### Outcome and selection of study population

The Norwegian studies by Mjoen et al. were published in 2012 [11] and 2014 [8] and report on a single centre experience with contradictory results (Table 1). They studied a consecutive cohort of 2269 donors who donated between 1963 and 2007 at a single centre in Oslo, Norway, where all kidney transplantations in Norway are performed. However, there were important differences in the selection and comparability of donors and non-donors (Tables 2, 3). In the 2014 study, 368 donors were excluded based on anti-hypertensive medication, blood pressure >140/90 mmHg, BMI > 30 kg/m^2^, age >70 years or <20 years, macroalbuminuria, or eGFR < 70 ml/min per 1.73 m^2^. This selection left only the healthiest donors. In the 2012 study, comparison data on non-donors were obtained from the Norwegian background population as provided by Statistics Norway. The 2014 study derived the comparison group from a Norwegian population-based cohort study (Helseundersøkelsene i Nord-Trøndelag, HUNT 1) carried out between 1984 and 1987 [14]. However, data on kidney function was not available for non-donors, while donors with low renal function were excluded from the analysis. Though similar donor and non-donor groups were studied, the other two research groups from the US and Canada reported on different outcomes, including long-term mortality [12], ESRD [9], death and major cardiovascular events [13], and gestational hypertension and preeclampsia [10]. The previous US analysis selected a donor cohort from the mandated national Organ Procurement and Transplantation Network (OPTN) registry. A total of 80,347 donors between 1994 and 2009 with a median follow-up of 6.3 years (maximum 12 years) were included in this study, excluding 36 donors for whom age was not recorded or were <18 years old. For the more recent US analysis the selection period was extended to 2011, increasing the donor cohort by 15,870 donors to a total of 96,217 donors with a median follow-up of 7.6 years (maximum 15 years). Both studies derived their comparison group of non-donors from National Health and Nutrition Examination Survey (NHANES) III participants [12]. NHANES participants were matched 1:1 to live donors with replacement to a predetermined maximum permissible radius. If information on the live donor’s BMI or systolic blood pressure was not available, a match was selected with healthy BMI (20–30 kg/m^2^) or systolic blood pressure (100–140 mmHg). Sampling with replacement was performed when a matched participant was the only fit despite ideal and radius matching. In the 2012 Canadian analysis, a donor cohort was selected from live kidney donors who donated between 1992 and 2009 in Ontario, Canada, and were permanent residents of Ontario [13]. The 2015 study included female live kidney donors who donated a kidney between July 1, 1992, and April 30, 2010, and who had at least one pregnancy with a gestation of at least 20 weeks during follow-up. The study population comprised only 88 donors. The non-donor comparison group for both studies was derived from the adult general population of Ontario in the Ontario Registered Persons Database, which contains demographic and vital status information for all Ontario residents. The starting date for follow-up was the date of nephrectomy and assigned as the index date. The donor index dates were randomly assigned to all adult residents of Ontario. Residents were excluded if any medical conditions that could preclude donation were known. For the 2015 study, in addition to previous restrictions depicted in Table 3, women with a previous diagnosis of gestational hypertension or preeclampsia were excluded from the analysis. Furthermore, the index data was extended to ±2 years to account for era effects. Each non-donor could be selected only once, resulting in 380,955 potential female non-donors (52 % of the original sample), though matched sets could be found for only 85 donors.

**Table 1:** Results of studies comparing live kidney donors to non-donors

Study	Year	Average follow-up, years	Outcome	Risk for donor	Overall results, donors versus non-donors	*p* value
Mjoen et al. [11]	2012	14.7	Overall mortality	↓	–	<0.001
			Cardiovascular mortality	↓	–	0.004
Mjoen et al. [8]	2014	15.1	All cause death	↑	HR 1.30 (95 % CI 1.11–1.52)	0.001
			Cardiovascular death	↑	HR 1.40 (95 % CI 1.03–1.91)	0.030
			End-stage renal disease	↑	302 cases per million; HR 11.38 (95 % CI 4.37–29.6)	0.001
Segev et al. [12]	2010	6.3	Long-term mortality	↓	1.5 versus 2.9 %	0.001
Muzaale et al. [9]	2014	7.6	End-stage renal disease	↑	30.8 per 10,000 (95 % CI 24.3–38.5) versus 3.9 per 10,000 (95 % CI 0.8–8.9)	0.001
Garg et al. [13]	2012	6.8	Death or major cardiovascular event	↓	2.8 versus 4.1 events per 1000 person years; HR 0.66 (95 % CI 0.48–0.90)	0.010
			Major cardiovascular event	↓	1.7 versus 2.0 events per 1000 person years; HR 0.85 (95 % CI 0.57–1.27)	–
Garg et al. [10]	2015	11.0	Gestational hypertension and preeclampsia	↑	11 versus 5 %; OR 2.4 (95 % CI 1.2–5.0)	0.010

*HR* hazard ratio, *CI* confidence interval, *OR* odds ratio

**Table 2:** Selection of live kidney donors and non-donors

Study	Year	Donors (n)	Donor participation (%)	Data collection	Non-donors (n)	Derived from	Average follow-up, years	Data collection
Mjoen et al. [11]	2012	2269	100	Norwegian Living Donor registry	6807	Norwegian background population	n.a.	Statistics Norway database
Mjoen et al. [8]	2014	1901	84	Norwegian Living Donor registry, Statistics Norway, Norwegian Renal Registry	32,621	HUNT 1 1984-1987	24.9	Survey database, Statistics Norway database, Norwegian Renal Registry
Segev et al. [12]	2010	80,347	100	OPTN registry, Social Security Death Master File	80,347	NHANES III	12.0	Survey database, Social Security Death Master File
Muzaale et al. [9]	2014	96,217	100	OPTN registry, CMS, deceased waitlist	96,217	NHANES III	15.0	Survey database, CMS
Garg et al. [13]	2012	2028	100	Medical records, Trillium database, CIHI-DAD, OHIP database, RPDB	20,280	General Canadian population	6.4	CIHI-DAD, OHIP database, RPDB
Garg et al. [10]	2015	85	97	Medical records, Trillium database, CIHI-DAD, OHIP database, RPDB	510	General Canadian population	10.9	CIHI-DAD, OHIP database, RPDB

*OPTN* Organ Procurement and Transplantation Network, *CMS* Centers for Medicare and Medicaid Services, *CIHI-DAD* Canadian Institute for Health Information Discharge Abstract Database, *OHIP* Ontario Health Insurance Plan, *RPDB* Ontario Registered Persons Database

**Table 3:** Comparability of live kidney donors to non-donors

Study	Year	Matched by	Statistics
Mjoen et al. [11]	2012	Restriction: Not performed	Kaplan Meier analysis
		Matching: 1:3 on age, gender, and year of birth	
Mjoen et al. [8]	2014	Restriction: Only inclusion of donors with a blood pressure ≤140/90 mmHg, BMI ≤30 kg/m^2^, no antihypertensive medication, age 20–70 years, no macroalbuminuria, and eGFR >69 ml/min per 1.73 m^2^. Only inclusion of non-donors with a blood pressure ≤140/90 mmHg, BMI ≤30 kg/m^2^, no diabetes or cardiovascular disease, no use of antihypertensive medication, and if participants rated their own health as “good” or “excellent”	Multiple imputation Coarsed exact matching
		Matching: on age, gender, year of inclusion, blood pressure, BMI, smoking	Cox regression
Segev et al. [12]	2010	Restriction of non-donors: Recorded kidney disease, diabetes, heart disease, and hypertension, and who had missing data on any of the four aforementioned criteria were excluded. The excluded participants also included those who answered positively to survey questions regarding “if doctors had told them that they had” heart disease, lupus, cancer, kidney stones or (pre)diabetes; difficulty independently performing physical activities or chest/leg pain while performing physical activities; or no health insurance because of poor health, illness, or age	Kaplan Meier analysis
			Log-rank test between group analysis
		Matching: 1:1 with replacement on gender, ethnicity, and history of cigarette smoking, and radius matching was done on age at donation, educational background, pre-operative BMI, and pre-operative systolic blood pressure	
Muzaale et al. [9]	2014	Restriction: No change in design	Kaplan Meier analysis
			Log-rank test within group analysis
		Matching: No change in design	Bootstrap methods between group analysis
Garg et al. [13]	2012	Restriction of non-donors: Evidence of diagnostic, procedural, or visit codes for genitourinary disease, diabetes, hypertension, cancer, cardiovascular disease, pulmonary disease, liver disease, rheumatological conditions, or chronic infections, a history of nephrology consultation, evidence of frequent physician visits (more than four visits in the previous two years), or any person who failed to see a physician at least once in the two years before the index date	Log-rank test
		Matching: 1:10 fashion on age (within two years), sex, index date (within six months), rural (population less than 10,000) or urban residence, and income (categorized into fifths of average neighbourhood income on the index date)	Cox regression
Garg et al. [10]	2015	Restriction: Only inclusion of donors who had at least one pregnancy with a gestation of at least 20 weeks during follow-up. No change in design for non-donors.	Generalized linear mixed model
		Matching: 1:6 fashion on age (within 2 years), sex, index date (within ±2 years), rural (population less than 10,000) or urban residence, and income (categorized into fifths of average neighbourhood income on the index date), the number of pregnancies carried to at least 20 weeks of gestation before index date (0, 1, or ≥2), and the time to the first birth after the index date	

### Data quality

Data for donors and non-donors were collected from pre-existing registries or databases (Table 2). Data were collected prospectively in national registries for live kidney donors in Norway, the US, and Ontario. In addition, the Canadian studies verified the donor data from Ontario’s central organ and tissue donation agency, the Trillium Gift of Life, with donor medical records from five major transplant centres. The Canadian studies did not state if there was any discrepancy between the donor registry and medical records. The outcomes were derived from registries in all six studies (Table 2). The Norwegian and Canadian studies, as well as the first US study in 2010, linked both the donor and non-donor data with the registries containing their studied outcomes. All outcomes were specifically coded within the registries. The recent US study in 2014 identified the outcome of ESRD differently for donors and non-donors, potentially leading to information bias. ESRD was defined as the initiation of maintenance dialysis, receipt of a living or deceased donor kidney transplant, or placement on the deceased waiting list. The outcome was ascertained by linkage to medical evidence Form 2728 for the Centers for Medicare and Medicaid Services (CMS). Donors were also linked to the transplant network’s kidney waiting list.

### Statistical analysis

All studies used both restriction and matching to address potential confounding except for the 2012 analysis by Mjoen et al. [11] (Table 3). The Norwegian research group added restriction and altered their matching method for their 2014 study. Mjoen et al. used Kaplan–Meier analysis without adjustment of confounders in 2012. In 2014, Mjoen et al. [8]. reported 31 ESRD events in 9 donors and 22 in non-donors. A majority of the donors who developed ESRD were immediate family members of the recipient. The Cox regression analyses for all outcomes including ESRD were adjusted for six confounders: age, gender, year of inclusion, blood pressure, BMI, and smoking. A second adjusted model was created after multiple imputation of blood pressure, BMI, and smoking. This latter model was used for the primary analyses. In contrast, the US and Canadian research groups did not alter the restriction and matching methods for their recent analyses. Although all outcomes were reported differently, as percentages, hazard ratios, or odds ratios depending on the statistical methods used. The US research group performed a Kaplan–Meier analysis in both studies but used a bootstrap method to properly estimate the variance of repeated sampling of non-donors in their most recent study [9]. The crude incidence of ESRD was 9 out of 1901 donors and 17 out of 32,621 non-donors, resulting in 36 cases of ESRD in the non-donor group after matching with replacement. Persons aged ≥65 years, African Americans, and Mexican Americans had an increased risk of ESRD, whereas Caucasian non-donors had no risk of ESRD. In the 2012 study by the Canadian research group [13], differences in baseline characteristics between donors and non-donors were assessed using standardized differences. If these differences were >10 % they would reflect a meaningful imbalance. A two-sided log-rank test stratified on matched sets was used to compare differences in death and cardiovascular outcomes between donors and non-donors. Furthermore, a Cox regression stratified on matched sets was used to estimate hazard ratios with 95 % confidence intervals. In the 2015 study by Garg et al. [10], generalized linear models with generalized linear estimating equations were used to compare the characteristics of donors and non-donors at the index date, and generalized linear mixed models with a random intercept and random effects logistic regression models were used to compare pregnancy characteristics and outcomes. These methods account for the correlation structure within matched sets and in women with more than one pregnancy during follow-up.

## Discussion

Our detailed review of the methodology of the different studies on long-term risk after live kidney donation revealed key differences with respect to the comparability of donors and non-donors in regard to selection, data quality, follow-up, and statistical analysis (Table 4).

**Table 4:** Overview of bias in selection of study population, data quality, and statistical analysis

Study	Year	Selection bias		Risk of donation	Information bias		Risk of donation	Confounding	Risk of donation
		Donors	Non-donors		Donors	Non-donors			
Mjoen et al. [11]	2012	−	+	Underestimation	+	−	Overestimation	−	n/a
Mjoen et al. [8]	2014	+	+	Overestimation	+	+	Overestimation	+	Overestimation
Segev et al. [12]	2010	−	+	Unclear	+	+	Overestimation	−	n/a
Muzaale et al. [9]	2014	−	+	Overestimation	+	+	Overestimation	+	Overestimation
Garg et al. [13]	2012	−	+	Underestimation	+	−	Overestimation	−	n/a
Garg et al. [10]	2015	−	+	No effect	+	−	Overestimation	−	n/a

+ or – bias present/not present in study

### Selection of the study population

Donors are a pre-screened healthy selection of the population. This is a key issue to account for when selecting the comparison group of non-donors. Furthermore, the extended donor selection criteria during the past decade [2] complicate restriction rules when including non-donors. Both Norwegian studies are a good example of choosing a more appropriate comparison group when studying the same donor population. In the 2012 study by Mjoen et al. [11], the full Norwegian background population was a comparison group without restriction according to the live kidney donor selection criteria. Therefore, the risk attributable to donation could be underestimated despite matching 1:3 on age, gender, and year of birth to account for confounding. In their 2014 study, Mjoen et al. [8], used the healthiest donors from the earlier study. In addition, more healthy non-donors were derived from a Norwegian population-based cohort study [14]. The restriction rules for donors and non-donors did not entirely lead to a match on renal function, cardiovascular disease, and subjective perception of health, leading to the possible overestimation of risk detrimental to donors because of healthier non-donors.

The US studies used more extensive restriction rules and matching for NHANES III participants compared to the healthier donors. NHANES III participants were derived from 81 counties in the US based on geography and the proportions of minority populations using probability proportionate to size sampling. Young children, persons aged ≥65 years, African Americans, and Mexican Americans were subgroups that were oversampled and were not representative of the donor population, the majority of which is Caucasian (75 %). Both studies used a similar restriction and matching strategy. The entire NHANES III cohort comprised 20,024 adult participants. The excluded group (n = 10,660) also contained participants who would be eligible for living donation, presumably making the non-donor group somewhat healthier than the donor population. The 9364 eligible NHANES III participants were significantly younger, more educated, had a higher proportion of women and Caucasians, and had a lower proportion of smokers than the donor population. This difference may have led to an overestimation of risk attributable to donation, which was however not demonstrated in the study by Segev et al.. The 2014 study by Mjoen et al. did demonstrated an increased mortality risk for donors. In the more recent US study, the strict selection of healthier non-donors made them less likely to develop ESRD. The donor population had significantly higher systolic blood pressure, BMI, and fraction of smokers at baseline, which are all factors associated with an increased risk of ESRD [15]. Thus, the risk attributable to donation was likely overestimated. In a recent study by Grams et al. [16], a proportion of the same aforementioned US donor population consisting of 52,998 live kidney donors was analysed based on their 15-year projected risk of ESRD, which was previously reported by Muzaale et al. [9]. In this recent study, the risk of ESRD among live kidney donors was compared to a meta-analysis of 4,933,314 participants in seven general population cohorts who would be eligible for living kidney donation according to 10 demographic and health characteristics. The average follow-up for these cohorts was 6.4 years and their 15-year risk projections for ESRD were compared among US live kidney donors. The donors had a 3.5–5.3-times higher projected 15-year risk than non-donors. As pointed out by Steiner [17], the previous US study by Muzaale et al. [9] reported an 8-times higher incidence of ESRD among donors than non-donors. This finding supports the notion that the risk attributable to donation was overestimated in that study.

Both Canadian studies used a similar restriction and matching strategy. The extended live donor eligibility criteria over the years have caused the broad exclusion criteria to encompass participants who would be eligible for living donation, making the non-donor group healthier. Furthermore, any person who failed to see a physician at least once in the 2 years before the index date was not included in the analysis in order to ensure that everyone who was included in the analysis had access to health care. This restriction could have led to the exclusion of the healthiest non-donors who did not require any medical attention in the past years and who would be highly eligible for living donation. Nevertheless, this exclusion criterion of healthier non-donors in the Garg et al. [10] study had no effect on the study results in a sensitivity analysis.

### Data quality

The strength of the data collection in all studies was that all data were collected mostly from national prospective registries. The Canadian studies even verified donor data with the donors’ medical records. However, there were some limitations in the data collection in regard to donor and non-donor medical outcomes and missing additional information on outcomes. Donors could be more aware of their health than non-donors, leading to differential misclassification because all outcomes except for death could have been registered earlier. This could have led to more registered outcomes among donors and an overestimated risk attributable to donation. Non-donor data from the population-based studies included data from surveys, giving a subjective rating of HUNT 1 and NHANES III participants’ health. These non-donor data were not verified with medical records, but were used for restriction, which could have led to an underestimation of risk among non-donors. The 2014 US study prioritized live kidney donors who developed ESRD on the deceased donor transplant waiting list [18]. Pre-emptively placing live kidney donors on the deceased waiting list possibly resulted in more registered donors with ESRD. This was seen in the higher crude incidence of ESRD among donors (99 out of 96,217) compared to non-donors (crude incidence 17 out of 9364). Non-donors who registered pre-emptively on the deceased waiting list were not identified as having ESRD, which caused a delay in the registration of ESRD for non-donors. However, their follow-up was longer than that of donors, and most non-donors would either receive a transplant or initiate dialysis shortly thereafter. Errors in the estimation of outcomes occurred in donors who emigrated; given the large sample sizes in both US studies, this is accepted to have had no material effect on the outcomes of the studies. Moreover, it will not affect the other studies given the high donor participation.

In the Garg et al. 2012 study, data on blood pressure is lacking [19], though previously the same authors demonstrated an increase in blood pressure [20], which increases the risk of cardiovascular events and mortality [21]. Lely et al. [22] pointed out that the severity and gestational age at which preeclampsia and gestational hypertension were diagnosed was not provided in Garg et al.’s 2015 study. Given that the rate of premature birth was not increased, only mild or at-term preeclampsia likely occurred [22]. Although there is an increased risk of preeclampsia and gestational hypertension in donors, the absolute risk is low and the severity of the complications, such as premature birth, are less than expected from a gynaecological point of view.

### Follow-up and statistical analysis

Differences between donors and non-donors in regard to comparability and follow-up should be accounted for during the analysis to overcome confounding. Restriction and matching is the first step, but matched sets and comparability should also be taken into consideration during the analysis. In Mjoen et al.’s 2014 study, the starting date of the follow-up for donors occurred decades earlier, causing an increased duration of follow-up, as pointed out by Boudville et al. [23], leading to a maximum follow-up time of 43.9 years for donors compared to a maximum of 24.9 years for non-donors. Boudville et al. suggested that secular changes in individuals’ health and health care made the baseline characteristics not fully comparable between the groups and could have resulted in a higher incidence of ESRD among donors. The authors tried to correct for this bias by adjusting for year of inclusion. Furthermore, Boudville et al. [23]. raised some concerns about statistical overfitting of the models used. For Cox proportional hazard models, a rule of thumb is to have at least 10 events per added confounder [24]. For the outcome ESRD, there were 31 reported events, but the primary analysis adjusted for six confounders. Both factors could have led to an overestimated risk attributable to donation. Furthermore, what stands out in the baseline characteristics of the donors and non-donors before any matching or adjustments were performed, as pointed out by Kaplan et al. [25], was the mean age difference of 46.0 ± 11.5 versus 37.6 ± 11.7 years, respectively. The higher age of donors could have been a plausible explanation for their increased risk of mortality. The Norwegian authors later replied that this difference was corrected by using coarsened exact matching in the survival analysis, which created strata of the potential confounders: age, gender, year of inclusion, blood pressure, BMI, and smoking. Donors and non-donors were matched based on these strata, after which the analysis was performed on non-coarsened data. After this matching the mean age of donors and non-donors was 46.0 versus 45.7 years, respectively [26]. For both US studies, Matuchansky [27] pointed out that a different NHANES cohort should have been selected instead of the participants from NHANES III; they proposed that participants in the “continuous NHANES” cohort beyond 1994, up to 2006, would have been a better chronological fit for their study cohort [27]. The US authors replied that the strength of NHANES III lies in its larger sample size, greater number of geographic areas, and availability of mortality linkage beyond 10 years. Furthermore, a limitation of “continuous NHANES” is that it cannot be used for survival comparisons [27, 28]. By using their specific bootstrap, the authors stated that this technique does not lead to bias, and differences in follow-up were accounted for by their use of survival analysis [29]. As pointed out by Gill et al. [30], in an editorial accompanying the study by Muzaale et al. [9], the crude incidence of ESRD was extremely low for NHANES participants: 17 out of 9364. Taken together with the longer follow-up of non-donors, replacement of non-donors with long event-free survival in matched analysis may have underestimated the risk of ESRD in non-donors [30]. The matching technique was also discussed by Matas et al. [15], who stated that matching with replacement could magnify any differences between donors and non-donors [15]. Furthermore, how many times each control was used was not stated. The authors replied that this technique has been established and that a specifically designed bootstrap was created to estimate the variance [31].

### Future perspectives

Live kidney donors are individuals who are not patients themselves, and submitting them to a surgical procedure stretches the Hippocratic oath taken by physicians. Although the absolute risks for donors following donation are very low, increased risks seem to exist among live kidney donors compared to non-donors. Risks both during and after donation are taken for granted by live kidney donors to help patients with ESRD [32]. Reduced risk of life-time dialysis, improved quality of life, and prolonged survival are gained by the recipients [1]. Furthermore, transplantation is far more cost-effective than dialysis [33, 34]. Nevertheless, these benefits for kidney transplant recipients should not outweigh the risks for live donors after donation. Therefore, future studies should focus on long-term outcomes following donation in which the risks for donors are taken into consideration against the risks for comparable non-donors.

## Conclusions

We conclude that recently published papers still face bias that could have led to a potential overestimation of risk attributable to donation. Even if risks are elevated among live kidney donors compared to non-donors, the absolute risks for donors following donation are very low and should therefore not discourage potential donors. Strong points of recent analyses compared to initial analyses are the extended time of follow up after donation, large sample sizes and better analysis, hence increasing the reliability to estimate potential risks for living kidney donors on the long-term. Key problems remain such as that donors are a pre-screened healthy selection of the general population, making it difficult to find an equal healthy unscreened comparison group. Specifically, not all required clinically relevant data are available for potential comparison groups. Selecting a healthier comparison group overestimates the risk attributable to donation. Future studies should focus on equal inclusion criteria for donors and non-donors, and in the analysis, follow-up duration, matched sets, and low absolute risks among donors should be accounted for when choosing the statistical technique. Ideally, long-term outcomes should uncover risk estimates for potential donors and how these risks would change if an individual becomes a live kidney donor.
